# Isolated Transverse Process Fractures: Should We Offer Lumbar Corset or Not?

**DOI:** 10.7759/cureus.57700

**Published:** 2024-04-06

**Authors:** Mustafa Kaya

**Affiliations:** 1 Department of Neurosurgery, Sakarya University Education and Research Hospital, Sakarya, TUR

**Keywords:** transverse process, corset, lumbar fractures, conservative, injury

## Abstract

Background

The aim of this study is to emphasize the need to be careful in terms of internal organ injuries in patients with isolated transverse process fracture (ITPF), and to investigate the effectiveness of corset use in controlling acute pain.

Methods

This is a retrospective study including 72 patients with only transverse process fractures secondary to trauma, who were admitted to the Emergency Department of Sakarya University Research and Training Hospital between January 2020 and October 2022. The radiological diagnoses were collected from spinal vertebral computed tomography images. Twelve patients were excluded from the study due to exclusion criteria. Sixty patients with ITPF were included in the comparison group. All patients were divided into two groups. The group with no lumbar corset (LC) included those who were discharged with analgesic and muscle relaxant treatment without a brace (n = 33). The LC+ group (n = 27) included those who received rigid lumbosacral orthosis in addition to analgesic and muscle relaxant treatment. Pain levels of all cases in both groups were evaluated with Visual Analog Scale scores on the day of trauma, the first week, the first month, and the sixth month.

Results

A total of 25 cases had one ITPF, 25 had two, 17 had three, and five patients had four or more ITPFs. The hospitalization rate was the highest among patients with four or more ITPFs (40%). Although the hospitalization rates according to the number of ITPFs were not statistically significant (p = 0.528), there was a clinical significance regarding increasing hospitalization rates with the increasing number of ITPFs. The hospitalization rates were 12%, 16%, 17.6%, and 40% in patients with one, two, three, and four or more ITPFs, respectively.

Conclusion

ITPFs should be treated conservatively. Concomitant organ injuries must be ruled out before treatment. Medical treatment without a lumbar corset could be used as a cost-effective choice.

## Introduction

As a term, isolated transverse process fractures (ITPFs) are defined as stable fractures that are not related to other anatomical structures surrounding the spine [1,2]. ITPFs are thought to be minor injuries compared to corpus, pedicle, and lamina fractures [3]. They can be seen at one or more levels in the lumbar vertebra and unilaterally or bilaterally. The most common cause in the young population is blunt high-energy trauma, such as motor vehicle accidents and sport-related collisions, and falling from height [1,4,5].

The mechanism of transverse process fracture (TPF) has been described to be a result of direct blunt trauma, violent lateral flexion-extension forces, avulsion of the psoas muscle, vertical shear of the pelvis, or high intra-abdominal pressures [6-8].

Although ITPF fractures are isolated fractures, the possibility of an abdominal injury should be considered. In this context, when this type of fracture is encountered, it is necessary to exclude visceral pathologies secondary to trauma [5,9,10].

ITPFs are stable fractures and most of the time, non-surgical treatment is recommended without neurosurgery consultation. Conservative treatment consists of analgesic treatment and orthoses such as collars or corsets with limited mobilization depending on the patient's ability to tolerate the pain [11-14]. Some studies have claimed that wearing a corset or other orthopedic orthosis is unnecessary [15,16].

Our study aims to highlight the two issues. The first is to emphasize the need to be careful in terms of internal organ injuries in patients with ITPF, and the second is to investigate the effectiveness of corset use in controlling acute pain.

## Materials and methods

Patient selection

Between January 2020 and October 2022, 72 patients with spinal trauma with lumbar transverse process fracture (LTPF) for whom neurosurgery consultation was requested from the Emergency Department of Sakarya University Training and Research Hospital were retrospectively examined. The radiological diagnoses were collected from spinal vertebral computed tomography images (Aquilion 64 CT Scanner, Toshiba, Tokyo, Japan).

Study inclusion criteria

Sixty patients with isolated TPF in the lumbar region without traumatic pathology in the cervical and thoracic vertebrae were included in the study.

Exclusion criteria for the study

Patients with spinal pathology who had vertebral body, pedicle, facet, lamina, spinous process fractures along with TPF in the lumbar region were not included in the study. In addition, 12 patients with internal organ injuries, extremity fractures, chest pathologies, and intracranial pathologies associated with LTPF were evaluated separately from the lumbosacral orthosis corset discussion as a hospitalization patient group.

Study design

A total of 33 patients were given analgesic and myorelaxant treatment without a rigid lumbosacral corset (RLSC) and were discharged (RLSC- group) whereas 27 patients were given an RLSC in addition to analgesic and myorelaxant treatment (RLSC+ group). The RLSC+ group was given the corset to be used while sitting and in an upright posture, other than lying down and resting. The duration of corset use was determined as six to eight weeks. The groups were evaluated with Visual Analog Scale (VAS) scores of 1 to 10 for back pain that were collected on the day of trauma, first week, first month, and sixth month.

Statistical analysis

Descriptive statistics were presented using mean and standard deviation values for continuous variables, and frequency and percent values for categorical variables. Comparisons between independent groups were done using the Mann-Whitney U test and chi-square tests for continuous and categorical variables, respectively. A type I error level of 5% was considered as a statistical significance threshold. SPSS version 25 (IBM Corp., Armonk, NY) was used for the analysis.

## Results

The mean age of the patients was 51.9 years, and 70.8% of them were males. The majority of patients had a primary etiology. The general characteristics of patients are presented in Table 1.

**Table 1:** General characteristics of the patients

	Mean ± SD
Age (years)	51.9 ± 17.7
	n (%)
Sex	
Male	51 (70.8)
Female	21 (29.2)
Etiology	
Fall	56 (77.7)
Motor vehicle accident (in-vehicle)	13 (18.1)
Motor vehicle accident (out-vehicle)	3 (4.2)

Clinical characteristics and hospitalization rates according to the number of ITPFs are presented in Table 2. A total of 25 cases had one TPF, 25 had two, 17 had three, and five patients had four or more TPFs. The hospitalization rate was the highest among patients with 4+ TPFs (40%). Although the hospitalization rates according to the number of TPFs were not statistically significant (p = 0.528), there was a clinical significance regarding increasing hospitalization rates with the increasing number of ITPFs. The hospitalization rates were 12%, 16%, 17.6%, and 40% in patients with one, two, three, and four or more ITPFs, respectively.

**Table 2:** Clinical characteristics and hospitalization rates according to the number of TPFs TPF: transverse process fracture. * Hospitalization rates according to TPF number were not statistically significant (p > 0.05).

TPF number	Frequency (patient)	The most commonly affected vertebra	Hospitalization-discharge	Hospitalization rate	^*^p
1	25 (34.7%)	40% L1	3-22	12%	0.528
2	25 (34.7%)	40% L1, L2	4-21	16%	
3	17 (23.6%)	70.6% L1, L2, L3	3-14	17.6%	
4+	5 (7%)	40% L1, L2, L3, L4	2-3	40%	

The comparison of the hospitalization rates according to the lateralization of the fractures revealed that the hospitalization rate was 40% (two of five cases) in bilateral cases but was only 14.9% (10 of 67 cases) in unilateral cases (Table 3). Although there was no statistically significant difference between unilateral and bilateral cases for hospitalization (p = 0.191), the rates were clinically significantly higher in bilateral cases (40% vs. 14.9%). Table 3 also summarizes the distribution of the departments where cases were hospitalized. Accordingly, of the unilateral cases, four patients were hospitalized in the thoracic surgery department with a diagnosis of hemothorax, three were hospitalized in the orthopedics department with the diagnosis of calcaneus fracture, radius fracture, and pelvic fracture, two were hospitalized in the general surgery department with the diagnosis of liver laceration, and one in the neurosurgery department with a diagnosis of traumatic sac.

**Table 3:** Hospitalization rates according to the lateralization of the fractures

Discharge-hospitalization	Lateralization		p
	Unilateral	Bilateral	
Discharge	57	3	0.191
Hospitalization	10	2	
Thoracic surgery	4	0	
Orthopedics	3	1	
General surgery	2	0	
Neurosurgery	1	1	

The comparison of VAS scores between patients with and without RLSC is presented in Table 4. Accordingly, the first day (p = 0.536), first week (p = 0.994), first month (p = 0.921), and sixth month (p = 0.899) VAS assessments were not significantly different between the groups.

**Table 4:** Comparison of Visual Analog Scales (VAS) scores between patients with and without rigid lumbosacral corset (RLSC)

Assessment	RLSC (+)	RLSC (-)	p
VAS 0	6.67 ± 1.03	6.55 ± 1.21	0.536
VAS 1^st^ week	2.30 ± 0.91	2.31 ± 0.98	0.994
VAS 1^st^ month	1.22 ± 0.95	1.22 ± 0.87	0.921
VAS 6^th^ month	0.36 ± 0.66	0.36 ± 0.68	0.899

## Discussion

It would be wrong to conclude that ITPFs are a rare fracture type due to the low reporting rates in the literature [2,9]. With the increase in the reporting of these cases in the literature and the widespread use of CT, they have become accepted as the most common type of fracture of the spine [5,17]. Most ITPFs are not fatal and conservative therapy is performed until the fracture is healed [10,18]. However, a lumbar TPF is not a simple minor trauma. Concomitant intra-abdominal visceral organ damage has been reported up to 48% in previous studies [12,19]. Thoracolumbar ITPFs are often accompanied by visceral injury (such as lung, liver, and kidney) [3,12,16,20].

Male predominance has been demonstrated in almost all studies for ITPF. In the present study, 70.8% of the cases were of males. It has been reported that women are more susceptible to visceral injury than men, regardless of the presence of ITPF. The reason for this has been attributed to some anatomical and physiological factors such as the distribution of adipose tissue in the body and the difference in bone mineral density [12,21]. In a single-center series, Akinpelu et al. [13] showed that the spine anatomically has sexual dimorphism, but this difference is unrelated to the transverse process. However, ITPF is higher in males, as they are generally more exposed to spinal trauma.

It has been emphasized in almost all publications about ITPFs that these fractures do not cause instability and neurological deficits [1,5,15,16]. No instability or neurological deficit was detected in any of the patients in the present study. Bradley et al. [12] concluded that there is no close relationship between the transverse process and the nerve root, based on the absence of any evidence of root damage in cases with ITPF. However, Alijanipour et al. [5] reported that they performed fusion surgery with posterior segmental instrumentation due to instability arising from soft tissue and ligament damage detected in the lumbar MRI of a patient who was determined to have ITPF on lumbar vertebra CT because of the lack of recovery of lumbar pain.

The lumbar region is the most common location for TPF. Multilevel TPF is more common in the lumbar region [1,2,5,9,15,16]. While childhood ITFPs are often seen at a single level, adult ITFPs are multilevel [2,11,15]. Multi-level ITFP cases generally appear to occur as a result of higher energy traumas than single-level cases. Moreover, these cases were accompanied by visceral injuries, which additionally caused serious mortality and morbidity [11,15]. Therefore, they claimed that as the number of fractures increased, the discharge of patients to rehabilitation centers or deaths increased. Gültekin et al. [4] stated that patients with four or more ITPFs and additional systemic injuries had a significantly greater rate of hospitalization, whereas those with one, two, or three ITPFs had a very low hospitalization rate, and a significant correlation was detected between the number of TPFs and the rate of hospitalization. In their study including athletes suffering from TPFs, Tewes et al. [19] observed a maximum of three TPFs with no additional pathology. Xia et al. [22] detected no relationship between the number of TPFs and additional pathologies.

The comparison of the hospitalization according to the lateralization of the fractures revealed that the hospitalization rate in bilateral cases was 40% (two of five cases), but in unilateral cases, this was only 14.9% (10 of 67 cases) (Table 3). Although there was no statistically significant difference between unilateral and bilateral cases for hospitalization (p = 0.191), the rates were clinically significantly higher in bilateral cases (40% vs. 14.9%). The statistical insignificance was associated with the low number of patients with bilateral fractures (n = 5). Table 3 also summarizes the distribution of the departments where cases were hospitalized because of various additional injuries.

Motor vehicle accidents rank first among the etiologies of ITPFs. In addition, falls (the second most common cause), blunt trauma (such as crushing), and bicycle and sports accidents can be counted among the causes [11,13,14]. Gültekin et al. [4] argued that there were two different types of TPF trauma mechanisms: (1) low-energy trauma (coronal injury, rear impacts) and (2) high-energy trauma (accompanied by visceral injury). Of the patients in the present study, 10 (13.88%) were exposed to high-energy trauma. While two of them were hospitalized in the general surgery clinic due to bland abdominal trauma and eight were exposed to coronal trauma by falling in various ways.

The morphology of ITPFs is linear fractures with or without avulsion or displacement [6]. The avulsion type of ITPFs has been shown to occur as a result of strong contraction of the musculus transversus abdominis (via the middle layer of the thoracolumbar fascia), musculus quadratus lumborum, musculus psoas muscles attached to the transverse process (TP) in the lumbar region, and the traction of L5 TP to the iliolumbar ligament [6,23-27]. Xia et al. [22] found a significant association between the L4 TP and abdominal injury. Because the middle layer of the thoracolumbar fascia L2 and L4 strongly attaches the TP, a strong contraction of this fascia first breaks L4, the thinnest transverse process. Xia et al. [22] suggested L4 TPF as a marker for abdominal injury. In the present study, one of two patients with intra-abdominal injuries had an L4 fracture and retroperitoneal bleeding. However, the other patient with liver contusion had only an L1 fracture. Previous studies have found a significant correlation between L5 TPFs and displaced pelvic fractures [25,28,29]. The L5 TP is attached to the iliolumbar ligament, which extends to the iliac crest [5]. It has been reported that L5 TPFs occur when this ligament is pulled due to a displaced pelvic fracture [23]. In the present study, two patients with dysplastic pelvis fractures had L5 TPF in line with the literature.

Gültekin et al. [4] found L1-L2 TPF association in 10 of 11 patients who were hospitalized for thoracic injury and considered this association as the avulsion fracture due to retraction of the lumbocostal ligament extending from the 12th rib to L1-L2 TPs. In the present study, the L1-L2 TPF association was found in all four patients who were hospitalized in the thoracic surgery department due to thoracic injury. The L2 TPF was found only in one of the two patients who were admitted to the orthopedic department due to the presence of orthopedic problems, while there was chest trauma and L1 and L2 TPF in the other patient. These findings were consistent with those of Gültekin et al. [4].

Previous publications have suggested that TPFs are stable injuries that do not cause neurologic deficits, and conservative treatment has been proposed for them [6,20]. Bradley et al. [12] argued that TPFs do not require any form of interventional surgery or bracing therapy. Moreover, they stated that they could be treated without the spine surgery (neurosurgery-orthopedics) consultation. Similarly, Boulter et al. [15] claimed that corset braces were not required for a successful recovery from ITPF and did not provide immobilization of the fracture, and the true benefit of bracing for patient comfort was likely limited. In fact, previous studies have discussed the negative impact of collars and halo vests on intracranial pressure and respiratory function, respectively [28,29]. Furthermore, braces can cause considerable discomfort and pose a potential cause of skin ulcers [13]. In some studies, it has been reported that the duration of hospital stay is prolonged with the treatment without a corset, the movements of the patients are restricted, and the recovery is delayed [11,13,15]. In the study by Gültekin et al. [4], 49 of 50 patients (98%) had a lumbosacral or thoracolumbar, with flexible support corsets applied. After corset application, severe pain and movement limitation decreased, breathing improved, and patients relaxed, and the use of a corset was recommended.

The use of nonsteroidal anti-inflammatory drugs (NSAIDs) in spinal pathologies affects the VAS scores in a positive way. Likewise, Beculic et al. indicated that preoperative use of NSAIDs as a conservative therapy for herniated disc-associated cauda equina syndrome affects the outcome assertively [30]. We found that publications in the literature on this type of spinal fracture have a common consensus regarding conservative treatment. This similarity is related to medical treatment targeting myofascial muscle damage and appropriate dosage of medication (anti-inflammatory drugs, muscle relaxants, and others) [1,2,5,15,16,30].

In the present study, NSAIDs and myorelaxant were initiated when all RLSC+ and RLSC- patients without concomitant organ injury were diagnosed. The comparisons of VAS scores between patients with and without RLSC are presented in Table 4. Accordingly, the baseline (p = 0.536), first week (p = 0.994), first month (p = 0.921), and sixth month (p = 0.899) VAS assessments were not significantly different between the groups. There are publications that support the use of corsets in providing pain control, as well as publications arguing that the use of a corset is ineffective in pain control and ITPF treatment. This is the first study to compare the two groups with and without a corset, despite the low number of patients. The present study has some limitations such as being retrospective, a limited number of patients, lack of standardization despite NSAIDs and myorelaxant treatment in all patients, that it is not a multicenter study, and the categories of the two centers compared are different (2nd and 3rd digit difference). ITFPs are stable fractures and do not need much medical attention after initial acute pain is controlled; therefore, the follow-up is difficult due to the tendency of patients to leave follow-up in the long term.

## Conclusions

Although there was a sufficient number of cases in this study, the study findings indicate that the VAS scores were similar between the two groups because a lumbar corset is not required for every patient. As emphasized in previous studies, corset treatment is not necessary in ITPFs.
